# Efficacy of Praziquantel for the Treatment of Human Schistosomiasis in Ethiopia: A Systematic Review and Meta-Analysis

**DOI:** 10.1155/2021/2625255

**Published:** 2021-12-20

**Authors:** Tamirat Hailegebriel, Endalkachew Nibret, Abaineh Munshea

**Affiliations:** ^1^Department of Biology, College of Science, Bahir Dar University, Bahir Dar, Ethiopia; ^2^Biotechnology Research Institute (BRI), Bahir Dar University, Bahir Dar, Ethiopia

## Abstract

**Background:**

Schistosomiasis is one of the neglected tropical diseases causing a serious human health problem in Ethiopia. Praziquantel is the only drug that has been used for the treatment of human schistosomiasis in the country. In line with this, the efficacy of praziquantel has been evaluated in a few interventional studies in the country, but there is a lack in systematically gathered and analyzed information for policymakers. The aim of this systematic review and meta-analysis was to provide a summary of the efficacy of praziquantel for the treatment of human schistosomiasis in Ethiopia.

**Methods:**

We conducted a literature search from ScienceDirect, PubMed/Medlin, and Google Scholar databases. A total of 140 articles published in English from 1980 to June 2021 were accessed and 15 of them were eligible for this meta-analysis. The meta-analysis was conducted using Stata 14 software, “metan command.” The heterogeneities among studies were evaluated using *I*^2^ test.

**Results:**

A total of 140 articles were reviewed, but only 15 of them fulfilled the inclusion criteria. The polled cure rate of 40 mg/kg praziquantel was 89.2% (95% CI: 85.4–93.1) and 93.6% (95% CI: 80.6–106) among *Schistosoma mansoni* and *S. haematobium*, respectively. Similarly, the mean egg reduction rates of 40 mg/kg praziquantel were 90.2% and 85% among *S. mansoni* and *S. haematobium* infected subjects, respectively. The common adverse events observed after receiving praziquantel include abdominal pain, vomiting, headache, diarrhea, and bloody stool.

**Conclusion:**

This systematic review and meta-analysis has indicated that praziquantel is still an appropriate drug for the treatment of human schistosomiasis in Ethiopia.

## 1. Introduction

Schistosomiasis is one of the neglected tropical diseases (NTD) which affects more than 230 million people in tropical and subtropical regions [1]. More than 90% of the cases are concentrated in African countries [2, 3]. Schistosomiasis is responsible for about 4.5 million disability-adjusted life years (DALYs) in endemic countries [4]. Human schistosomiasis is caused by six *Schistosoma* species. Among these, *Schistosoma mansoni, S. haematobium*, and *S. japonicum* are responsible for 99% of human schistosomiasis [5], of which the first two are the predominant and widely distributed species [6, 7], while *S. japonicum* is restricted in China, Indonesia, and parts of the Philippines [8].

In Ethiopia, estimates indicate that about 37.5 million people are living at risk of schistosomiasis, of which more than 5 million people are chronically infected [9]. Schistosomiasis is one of the major causes of outpatient morbidity in the country [10]. *Schistosoma mansoni* is the most common cause of human intestinal schistosomiasis followed by *S. haematobium,* which is a cause of urogenital schistosomiasis in the country. Although there is no nationwide survey, several epidemiological studies have indicated the existence of a moderate prevalence of schistosomiasis in the country. The prevalence of *S. mansoni* ranged from 10% to 92%, while *S. haematobium* ranged from 5% to 58% in the country [11]. High prevalence of schistosomiasis has been reported among school-aged children in the country [12, 13].

Although intensive research has been carried out on the development of a vaccine against schistosomiasis, there is no licensed vaccine yet. Thus, the available option is the use of praziquantel for the treatment of infected cases in many endemic countries since the 1970s. Besides praziquantel for treatment and a future vaccine for prevention, there are at least three other mainstems for tackling schistosomiasis: snail control, sanitation, and health education. Praziquantel is a relatively safe, well-absorbed, and effective oral drug that is active against all schistosome species [14]. The recommended dose is 40 mg/kg body weight and it is taken orally as a single dose (40 mg/kg), or this dose is divided over a day (2 × 20 mg/kg doses every 4 hours) [15] for the treatment of human schistosomiasis. Similarly, 60 mg/kg dose of praziquantel is also recommended for the treatment of human schistosomiasis for children [16, 17]. The drug usually acts within one hour of ingestion by paralyzing the worm and damaging the tegument. However, praziquantel has little or no effect on eggs and on immature worms [18]. In addition, repeated usage of praziquantel for the treatment of infected individuals and its mass drug administration among school children raise concerns about the efficacy of the drug in endemic countries. As a result, poor treatment outcomes of praziquantel were reported from several countries [19–21].

Praziquantel is widely used for the treatment of patients as well as mass drug administration (MDA) among school-aged children in Ethiopia. Limited studies are available on the efficacy of praziquantel in the country. The standard approach to assess drug efficacy was by determining the cure rate (CR) after administration of the standard dose of praziquantel. WHO has recently recommended egg reduction rate (ERR) as another approach to assess the efficacy of anthelminthic drugs [22]. CR is defined as the proportion of schistosome infected individuals who become parasitologically negative after administration of the standard dose (e.g., a single dose, 40 mg/kg) of the drug. ERR is equal to 1- (arithmetic mean of egg count after treatment divided by arithmetic mean egg count before treatment) expressed in percentage. Although quite limited and fragmented interventional studies are available in the country, to the best of our knowledge, there has not been any summarized data up to this point in time on the efficacy of praziquantel at the national level. Therefore, this systematic review and meta-analysis aimed to systematically summarize the existing fragmented efficacy studies in the country.

The PICOS (patients, interventions, comparisons, outcomes, and study design) framework used in this review is described as follows: P, schistosome infected individuals; I, administration of single-dose 40 mg/kg praziquantel; C, no comparisons; O, schistosome infection cure-rate and egg reduction rate; and S, observational studies.

## 2. Methods

### 2.1. Search Strategies

We conducted a literature search from online public databases, mainly from PubMed/Medline, Google Scholar, and ScienceDirect in June 2021. The selection was done using the following terms and Boolean operators: “Schistosomiasis” OR “praziquantel efficacy” OR “*S. mansoni*” OR “*S. haematobium*” AND “Ethiopia.” All articles published between 1980 and June 2021 in English were included in this meta-analysis. The systematic review and selection of relevant literature were conducted according to PRISMA (Preferred Reporting Items for Systematic Reviews and Meta-Analysis) guidelines [23] (Table S1).

### 2.2. Inclusion and Exclusion Criteria

Literatures published in the English language were extracted from online public databases. Only original articles published in peer review journals report the efficacy of praziquantel among Ethiopian populations. All articles that were interventional studies (observational studies) that administered single-dose praziquantel at 40 mg/kg body weight were included in the list of eligible articles. Articles that lacked information about cure rates or detailed information about study subjects and review papers and studies conducted on nonhuman subjects were excluded from this meta-analysis.

### 2.3. Data Extraction Protocol

The data extraction protocol was developed by TH and reviewed by EN and AM. The data extraction protocol consisted of the name of authors, year of publication, study region, sample size (population), population type (community or outpatient or school or preschool), age range, diagnostic methods, *Schistosoma* species targeted, cure rate praziquantel (number of cured/number of positive subjects), egg reduction rate, follow-up time, side effects of the praziquantel, and purpose of intervention (treatment or prevention).

### 2.4. Quality of Individual Study

The quality of individual studies was assessed using Quality Assessment Tools for interventional studies [24]. Individual studies were assigned a score of either No (0) or Yes (1) for the ten parameters that were formulated based on the objective of the review. The quality was determined by counting the number of Yes (1) scores in each of the ten parameters. The overall quality of the individual study was classified as low quality (scores of 1–4), moderate quality (scores of 5–7), and high quality (scores of 8–10).

### 2.5. Risk of Publication Bias

The risks of publication bias across studies were assessed using funnel plot symmetry (qualitative estimation) and Egger's regression test and Begg's rank correlation test (quantitative estimation).

### 2.6. Intervention and Outcomes

Schistosomiasis caused by *S. mansoni* and *S. haematobium* was included in this meta-analysis. The participants involved in the interventional studies were outpatients, school children, preschool children, and community-based studies of any age. The participants of all the interventional studies received the WHO recommended 40 mg/kg body weight single-dose praziquantel for the treatment of schistosomiasis. The outcome of the evaluation was a parasitological cure, which was defined as eggs-positive or eggs-negative or absence of symptoms associated with schistosomiasis.

### 2.7. Data Analysis

Cure rate was determined by dividing the number of cured individuals by the total positive subjects before treatment as indicated in each article. The confidence intervals for the cure rate were set at 95%. The egg reduction rates were obtained from the articles and calculated as the arithmetic mean. All studies with a follow-up period of two to eight weeks were included in the meta-analysis. Subgroup analyses were conducted based on a region of study, nature of participants, *Schistosoma* species, and years of study. The random-effects model was used to combine the pooled cure rate due to the presence of heterogeneity among studies at 95% CI. Egger's regression test and Begg's rank correlation test (quantitative) and funnel plot (qualitative) were used to assess the presence of publication bias. Metaregression analyses were performed to assess the sources of heterogeneity. The meta-analysis was performed using Stata software (version 14, STATA Corp, College Station, TX), where *p* < 0.05 was considered as statistically significant.

## 3. Results

### 3.1. Selected Studies

**Figure 1:**
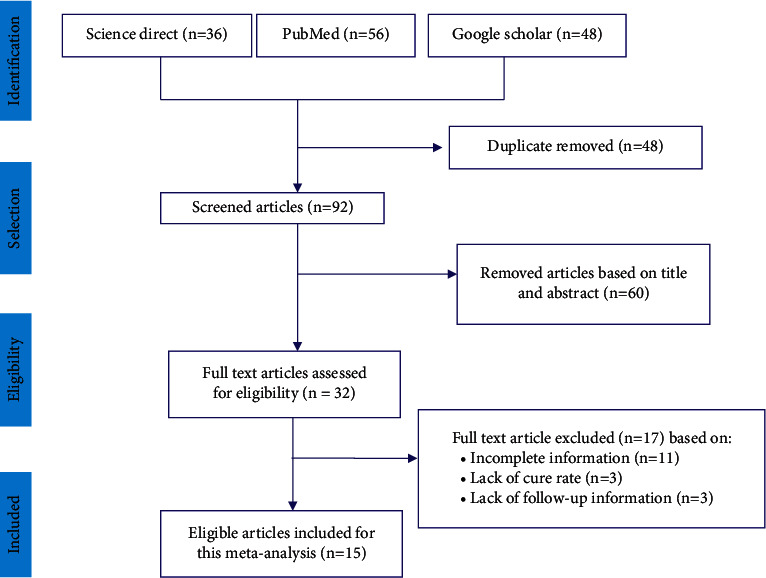
PRISMA flow diagram showing the selection process of articles used to determine the efficacy of praziquantel among the Ethiopian population.

From the total of 140 articles available online in public databases, 48 articles were excluded due to duplicates. Of the remaining 92 screened articles, 60 were excluded after we reviewed their titles and abstracts. The remaining 32 full-text articles were accessed for eligibility based on the inclusion criteria and information indicated in the data extraction protocol. As a result, 17 articles were further excluded in the data extraction process primarily due to the outcome of interest, having no cure rate, and lack of clear figures on the total number of study participants and treated cases. Only 15 articles [25–39] fulfilled the eligibility criteria and were included in this systematic review and meta-analysis (Figure 1).

### 3.2. Study Characteristics

The studies included in this meta-analysis were obtained from six regional states of Ethiopia, namely, Amhara, Oromia, Somali, South Nations and Nationalities Peoples (SNNP), Tigray regions, and Addis Ababa city administration. All eligible articles that were nonrandomized interventional studies were used in this meta-analysis. All interventional studies that used 40 mg/kg single-dose praziquantel with a follow-up period ranging from 2 to 8 weeks were included in the analysis. Detailed information about the characteristics of the articles included in this meta-analysis is shown in Table 1.

### 3.3. Risk of Bias within Studies

We assessed the quality of the individual study using Quality Assessment Tools for interventional studies. The quality score obtained from the individual study was moderate and high. The overall analysis showed that there was a low risk of publication bias within studies.

### 3.4. Pooled Efficacy of Praziquantel for the Treatment of Schistosomiasis

**Figure 2:**
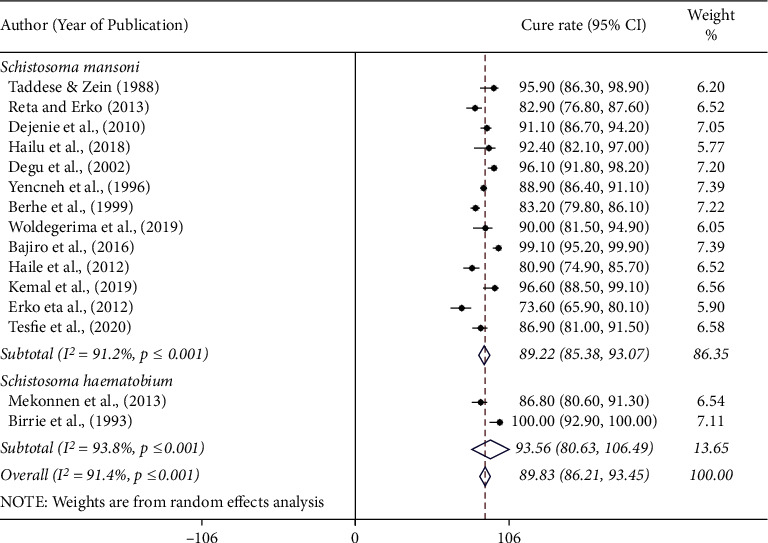
Forest plot showing the cure rate of praziquantel at 40 mg/kg for the treatment of human schistosomiasis based on *Schistosoma* species in Ethiopia.

The efficacy of praziquantel was assessed using the cure rate and egg reduction rate among schistosome infected subjects. The cure rate of 40 mg/kg single-dose praziquantel was ranged from 73.6% to 100% among schistosome infected individuals in Ethiopia (Table 1). The pooled cure rate of a single dose of 40 mg/kg praziquantel was 89.8% (95% CI: 86.2–93.5) for human schistosomiasis. The pooled cure rates of praziquantel were 89.2% (95% CI: 85.4–93.1) and 93.6% (95% CI: 80.6–106) among *S. mansoni* and *S. haematobium* infected individuals, respectively (Figure 2). However, twelve of the total fourteen studies were carried out on *S. mansoni*, while only two studies were conducted on *S. haematobium* in the country.

Egg reduction rate was the second parameter used to assess the efficacy of praziquantel treatment. The ERR among *S. mansoni* infected individuals ranged from 79.5% to 99.6%, while ERR was 85% among *S. haematobium* infected individuals. Unfortunately, 5 out of the 15 eligible articles (33.3%) did not report ERR among treated individuals.

### 3.5. Publication Bias Across Studies

**Figure 3:**
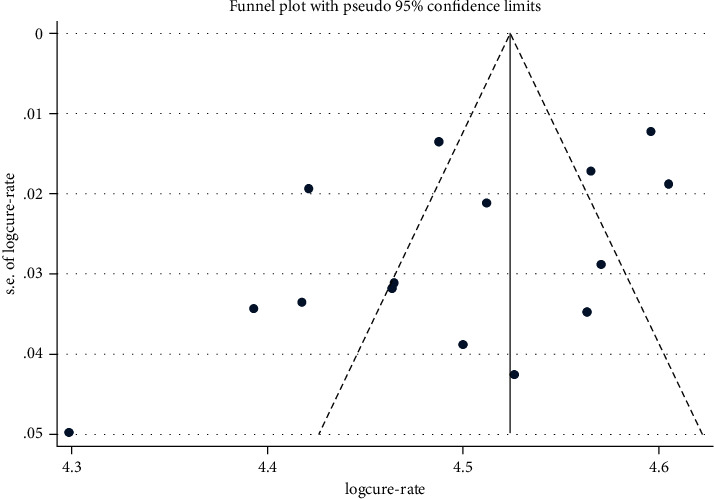
Egger's funnel plot indicating possible publication bias on the efficacy of 40 mg/kg praziquantel for the treatment of human schistosomiasis.

The funnel plot symmetry indicated that there was no publication bias across studies (Figure 3). Similarly, Egger's test (*p* value = 0.069) and Begg's test (*p* value = 0.138) confirmed the absence of publication bias among studies included in this meta-analysis.

### 3.6. Subgroup Analysis

**Figure 4:**
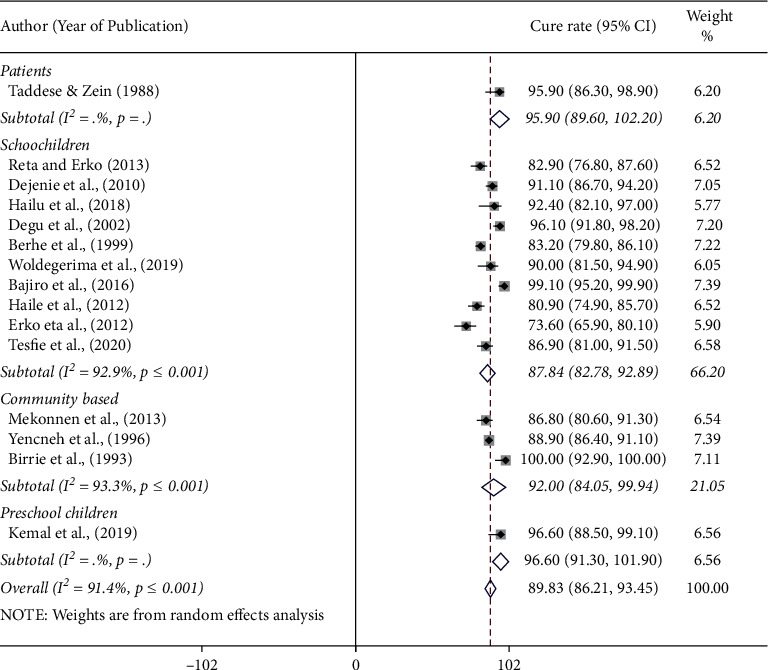
Forest plot showing the cure rate of praziquantel at 40 mg/kg for the treatment of human schistosomiasis based on the nature of study participants in Ethiopia.

**Figure 5:**
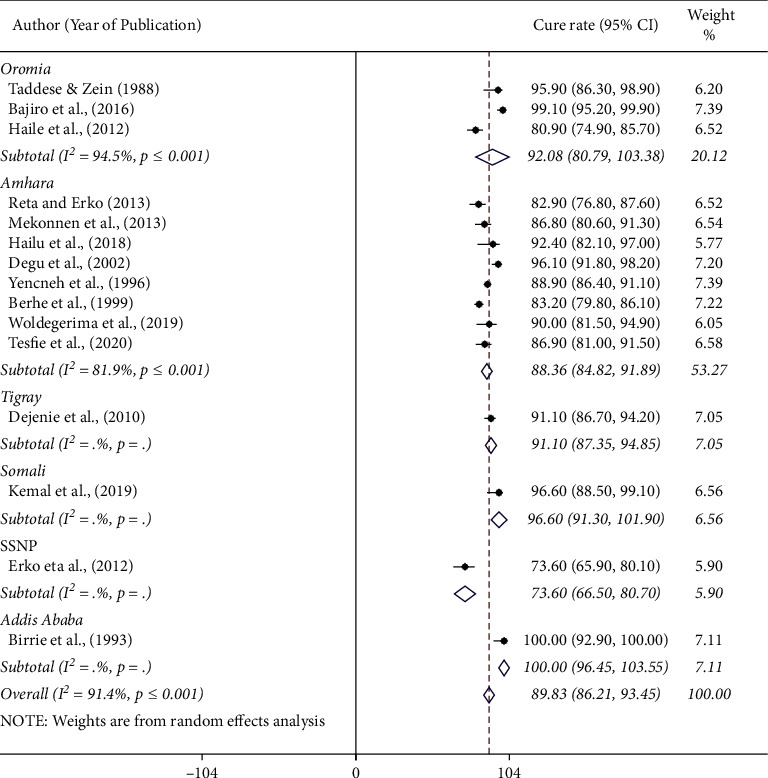
Forest plot showing the cure rate of praziquantel at 40 mg/kg for the treatment of human schistosomiasis in Ethiopia by region of study.

**Figure 6:**
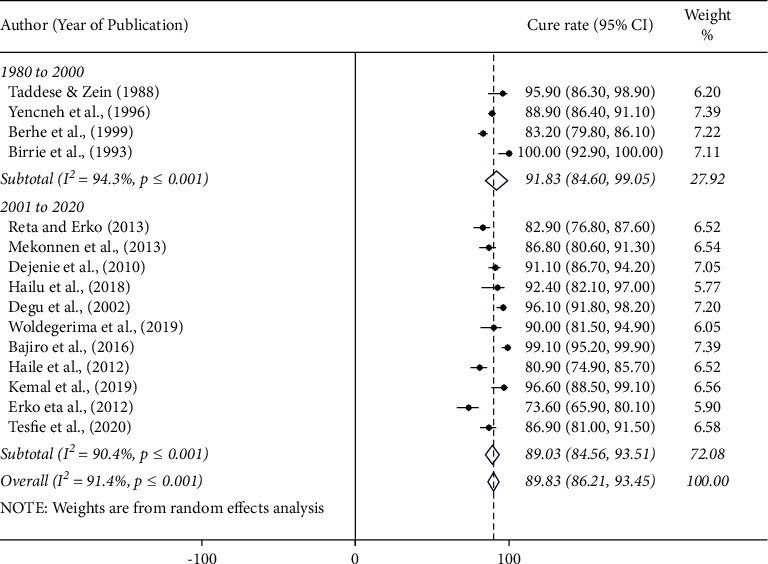
Forest plot showing the cure rate of praziquantel at 40 mg/kg for the treatment of human schistosomiasis based on years of studies in Ethiopia.

The pooled cure rate of praziquantel was the highest, 96.6% (95% CI: 88.5%–99.1%) among preschool children followed by 95.9% (95% CI: 86.3%–98.9%) among patients, 91.6% (95% CI: 89.8%–93.5%) in community based studies, and 87.8% (95% CI: 82.8%–92.8%) among schoolchildren (Figure 4). There was no significant difference in the efficacy of praziquantel against human schistosomiasis among regions in Ethiopia. The pooled cure rate of praziquantel treatment was 88.4% (95% CI: 84.8–91.8) in the Amhara region and 92.1% (95% CI: 80.8–100) in the Oromia region. The results of the efficacy study from Addis Ababa, Afar, Tigray, and SNNP regional states (single efficacy study from each region) are presented in Figure 5. We assessed the possibility of efficacy variation with respect to years of study (1980 to 2000 or 2001 to 2021). The pooled efficacy of praziquantel was 91.8% (1980 to 2000) and 89.0% (2001 to 2021) in the country (Figure 6).

### 3.7. Metaregression and Sensitivity Analysis

The result of this meta-analysis showed the presence of heterogeneity among studies (*I*^2^ > 90%). We performed a regression analysis to identify the sources of heterogeneity across studies included in this meta-analysis. The regression coefficient for several variables is as follows: region of study (1.007, 95% CI: 0.978–1.038, *p*=0.596), nature of study participants (1.037, 95% CI: 0.987–1.089, *p*=0.138), year of publication (0.970, 95% CI: 0.874–1.078, *p*=0.548), and *Schistosoma species* (1.049, 95% CI: 0.916–1.202, *p*=0.459). However, these variables did not contribute to the observed heterogeneity. Sensitivity analysis was performed by excluding one interventional study at a time and calculates the pooled CR. The result of this analysis did not bring any significant change to the pooled CR.

### 3.8. Adverse Events

Adverse events were observed among 1079 (88.7%) individuals who had taken praziquantel for the treatment of human schistosomiasis (Table 2). Adverse events of praziquantel were presented from 7 (50%) of the interventional studies included in this review. The common adverse events include abdominal pain, headache and vomiting [26, 29, 31–34, 36], diarrhea [26, 29, 32–34, 36], bloody stool [26, 29, 32, 33, 36], nausea [26, 29, 31–33], dizziness [26, 33, 34, 36], fatigue [29, 31–33], fever [29, 31–33], and drowsiness [29, 32, 33]. These signs and symptoms were observed for the first four hours after treatment and resolved shortly.

## 4. Discussion

Praziquantel is the only drug available for the treatment of human schistosomiasis in many endemic countries. Praziquantel has been used for the treatment of human schistosomiasis for more than forty years. Several interventional studies on the efficacy of the praziquantel are available in Ethiopia. Therefore, summarized data is needed to evaluate the overall efficacy of a single dose of 40 mg/kg praziquantel for the treatment of human schistosomiasis in the country.

This meta-analysis showed that a single dose of praziquantel has 89.8% cure rate for the treatment of human schistosomiasis in Ethiopia. In line with our findings, a higher cure rate of a single dose of 40 mg/kg praziquantel was reported in several countries [40–43]. In contrast, a lower cure rate of praziquantel was reported from a meta-analysis elsewhere [44, 45]. The present study revealed that a single dose of 40 mg/kg praziquantel is effective against human schistosomiasis in Ethiopia. On the contrary, a repeated dose of 40 mg/kg praziquantel had a higher cure rate than a single dose of praziquantel [17, 46]. Moreover, 60 mg/kg praziquantel was more effective than the standard 40 mg/kg single-dose praziquantel for the treatment of human schistosomiasis [47]. These variations of the efficacy of praziquantel might be associated with baseline infection intensity, nature of study population, sample size, and brand of the drug used.

The efficacy of praziquantel was slightly higher among *S. haematobium* infected patients than among *S. mansoni* infected cases. However, only two studies were conducted on the efficacy of praziquantel against *S. haematobium* from the total twelve studies included in this meta-analysis. *Schistosoma haematobium* is generally restricted in lowland (below 800 masl) areas [11, 31, 48, 49] and only a few studies are available in the country. Therefore, it is not wise to compare the efficacy of the drug against the two schistosome species based on the existing information. Similar to our finding, a higher cure rate of praziquantel was reported from preschool and schoolchildren infected with *S. haematobium* compared to those infected with *S. mansoni* [44, 47, 50]. In contrast, a higher cure rate of praziquantel was reported among individuals infected with *S. mansoni* than those infected with *S. haematobium* in Cameroon [51]. The difference in the efficacy of praziquantel might be associated with the endemicity of the disease in the area, baseline infection intensity, age of study participants, and presence of immature stages in addition to the inherent biological difference of species.

The cure rate of a single dose of 40 mg/kg praziquantel was slightly lower in the Amhara region than in other regional states in Ethiopia. This might be associated with the high endemicity of the disease in the Amhara region. Several studies indicated high prevalence (>83%) of *S. mansoni* in the region [12, 13, 38]. The high prevalence and intensity of infection may contribute to the slightly lower efficacy of praziquantel in the region. In line with the high infection intensity, there may be large number of immature worms, which is insensitive for the drug that contributes to lower efficacy in the region. Moreover, 8 (53.3%) of the interventional studies included in this meta-analysis were derived from the Amhara region. There were only four studies reported from Tigray, SNNP, Somalia, and Addis Ababa city administrations (one study from each regional state). As the number of studies increased, there might be a variation in their methodology, follow-up time, level of endemicity, nature of the study population, and sample size. These variations may lead to differences in the efficacy of praziquantel among interventional studies which might contribute to the slightly reduced cure rate in the region.

The efficacies of praziquantel were almost similar in all types of study participants (patients, schoolchildren, preschool children, and the community). The efficacy of praziquantel was slightly higher among preschool children than among schoolchildren as reported elsewhere [44]. In this meta-analysis, only one study reported the efficacy of praziquantel from preschool children in Ethiopia. It might not be good to compare the efficacy of praziquantel between schoolchildren and preschool children. Similarly, we compared the cure rate of praziquantel from 1980 to 2000 and from 2001 to 2021 to assess the possibility of variation in response rate due to frequent exposure. The result showed that the cure rate is slightly lower in recent years than in studies reported before 2000. However, the difference is not statistically significant. This shows the absence of drug failure and/or resistance against praziquantel, suggesting that the drug is still effective in the treatment of schistosomiasis in Ethiopia.

The study showed high heterogeneity (*I*^2^ = 91.4%) across studies. We conducted a metaregression analysis to assess the source of heterogeneity by considering region of study, nature of study participants, year of study, and *Schistosoma* species. All these did not contribute to the observed heterogeneity. Other conditions such as sample size, infection intensity, and specific intervention time and area might contribute to the observed heterogeneity across studies.

Egg reduction rate was the second recommended option to assess the efficacy of anthelminthic drugs in endemic countries [22]. The egg reduction rate after praziquantel administration reported among studies included in this meta-analysis ranged from 68.2% to 99.6%. The pooled egg reduction rate was 89.6% among studies that reported ERR. Praziquantel contributes to more than 90% of ERR of schistosome infection as reported elsewhere [41, 45, 52]. Anthelminthic drugs that reduce the infection intensity at least by 90% are accepted and are recommended to continue for the treatment of human schistosomiasis [22]. The studies included in this meta-analysis used the standard 40 mg/kg single-dose praziquantel. Studies showed that a double-dose arm of 40 mg/kg of praziquantel resulted in a higher ERR compared to the standard single-dose arm [17, 46].

About 7/14 (50%) of the interventional studies reported the presence of adverse events of praziquantel within 4 hours after drug administration, but all adverse events were subsided within a short period of time. The most common adverse events including abdominal pain/discomfort/cramp, headache, and vomiting followed by diarrhea, nausea, itching, bloody stool, fever, fatigue, and dizziness were reported in at least four studies from the total seven studies that reported adverse events of praziquantel. Similar adverse events of praziquantel were reported in systematic review and meta-analysis from schistosomiasis endemic countries [44, 45]. The adverse events might be associated with the host immune response to the parasite antigens released from the dead parasites. In addition, the intensity and location of the infection may also contribute to the adverse side effects.

About 89% of the treated individuals showed one or more adverse events of praziquantel observed in study subjects included in this review. Similar high adverse events of praziquantel were reported from Brazil [53] and Uganda [54]. In contrast to our findings, a lower adverse event of praziquantel was reported elsewhere [43, 45, 50]. These differences might be associated with the difference in infection intensity, parasite, or host factors.

## 5. Limitation of the Study

This meta-analysis summarized the praziquantel efficacy studies carried out in the country and produced pooled cure rates and egg reduction rates. Nevertheless, it has some limitations. First, the studies included in this meta-analysis lack a control group or individuals taking a placebo during the intervention time. This makes it is impossible to calculate risk ratio (RR) or relative risk. Therefore, we were forced to use the cure rate rather than the risk ratio. Second, the studies eligible for this meta-analysis used different parasitological methods (Kato-Katz, urine filtration, and formol-ether concentration). These methods have different sensitivity and specificity that may affect the outcome of praziquantel efficacy. It is known that Kato-Katz and urine filtration methods are needed for the diagnosis of *S. mansoni* and *S. haematobium* infection, respectively. Third, egg reduction rate was not available in all articles included in this meta-analysis. Fourth, the adverse side effects of praziquantel were not reported from all eligible articles for this study.

## 6. Conclusion

This systematic review and meta-analysis revealed that praziquantel is still an effective drug for the treatment of human schistosomiasis in Ethiopia. A single dose of 40 mg/kg praziquantel administration resulted in >90% in both cure and egg reduction rates in Ethiopia. Mild and transit adverse effects including abdominal pain/discomfort, headache, vomiting, diarrhea, bloody stool, nausea, and itching were consistently observed among individuals after receiving the treatment but resolved within 24 hours. This meta-analysis supports the use of a single dose of 40 mg/kg praziquantel for the treatment of patients as well as for mass drug administration. Praziquantel treatment should be supported by environmental sanitation and proper health education for effective control and elimination of human schistosomiasis in Ethiopia.

## Figures and Tables

**Table 1:** Summary of the characteristics of eligible studies included in this meta-analysis for evaluating praziquantel drugs used for the treatment of human schistosomiasis in Ethiopia.

Author, year	Study area (s)	Region	Participants	Age (years)	Schistosome species	Diagnostic approach	Follow-up time	Total population	Cured/treated	Cure rate (%) (95% CI)	ERR (%)
Taddese and Zein, 1988	Wonji	Oromia	Patients	17–52	*S. mansoni*	Kato-Katz	4 weeks	200	47/49	96 (86.0–99.5)	90
Birrie et al., 1993	Addis Ababa	Addis Ababa	Community based	20–34	*S. haematobium*	N/A	6 weeks	50	50/50	100 (92.9–100)	N/A
Yencneh et al., 1996	Jiga	Amhara	Community based	>5	*S. mansoni*	Kato-Katz	8 weeks	4861	596/670	89 (86.5–91.3)	N/A
Berhe et al., 1999	Bati, Harbu, and Kemise	Amhara	Schoolchildren	5–17	*S. mansoni*	Kato-Katz	5 weeks	611	450/541	83.2 (80–86.3)	N/A
Degu et al., 2002	Gorgora	Amhara	Schoolchildren	10–14	*S. mansoni*	Kato-Katz	6 weeks	325	148/154	94 (93.0–99.1)	97
Dejenie et al., 2010	Tumuga and Waja	Tigray	Schoolchildren	5–19	*S. mansoni*	Kato-Katz	4 weeks	390	205/225	91.1 (87.4–94.8)	N/A
Reta and Erko, 2010	Senbete Town	Amhara	Schoolchildren	10.81	*S. mansoni*	Kato-Katz	4 weeks	342	155/187	82.9 (77.5–88.3)	79.5
Erko et al., 2012	Wondo Genet	SNNP	Schoolchildren	6–22	*S. mansoni*	Kato-Katz	4 weeks	299	106/144	73.6 (65.9 –80.1)	68.2
Haile et al., 2012	Finchaa Valley	Oromia	Schoolchildren	6–14	*S. mansoni*	Kato-Katz	4 weeks	324	165/204	80.9 (75.5–86.3)	99.5
Mekonnen et al., 2013	Blue Nile Vally	Amhara	Community based	2–60	*S. haematobium*	Urine filtration	7 weeks	341	132/152	86.8 (81.5–92.2)	85
Bajiro et al., 2016	Mana district	Oromia	Schoolchildren	6–18	*S. mansoni*	Kato-Katz	3weeks	500	114/115	99.1 (95.2–99.9)	99.9
Hailu et al., 2018	Bahir Dar	Amhara	Schoolchildren	6–14	*S. mansoni*	FECT	2 weeks	409	49/53	92.4 (85.3–99.5)	N/A
Kemal et al., 2019	Erer Health Center	Somali	Preschool children	<6	*S. mansoni*	Kato-Katz	4 weeks	236	57/59	96.4 (88.3–99.5)	99.4
Woldegerima et al., 2019	Sanja Town	Amhara	Schoolchildren	9–14	*S. mansoni*	Kato-Katz	3 weeks	372	72/80	90 (83.4–96.5)	99.6
Tesfie et al., 2020	Sanja Town	Amhara	Schoolchildren	6–18	*S. mansoni*	Kato-Katz	4 weeks	245	153/176	86.9(81.0–91.5)	78.3

**Table 2:** Adverse side effects observed among individuals treated with 40 mg/kg praziquantel in Ethiopia.

Adverse events reported	No. of studies	Patients treated	Treated patients showing AEs	Incidence (%)	95% CI	
					Lower limit	Upper limit
Abdominal pain/cramp	7	1217	842	69.2	66.5	71.8
Diarrhea	6	1158	419	36.2	33.4	39.0
Headache	7	1217	345	28.3	25.8	30.9
Nausea	5	1066	317	29.7	27.0	32.6
Vomiting	7	1217	305	25.1	22.6	27.6
Dizziness	4	839	237	28.2	25.2	41.4
Bloody stool	5	1108	184	16.6	14.5	18.9
Body weakness	2	724	157	21.7	18.7	24.9
Fever	4	537	138	25.7	22.1	29.6
Fatigue	4	537	126	23.5	19.9	27.3
Straining	2	283	116	40.9	35.2	46.9
Itching	5	629	89	14.1	11.5	17.1
Drowsiness	3	478	88	18.4	15.0	22.2
Haematuria	1	65	65	98.5	91.7	100.0
Epigastric pain	2	694	63	9.1	7.0	11.5
Dysuria	2	283	58	20.5	15.9	25.7
Stomachache	1	59	10	16.9	8.4	28.9
Dermatological code	1	101	9	8.9	4.2	16.2
Sweating	1	59	4	6.8	1.9	16.5
Body/skin rash	1	195	3	1.5	0.3	4.4
Swelling	1	195	3	1.3	0.3	4.4
Any one of these AEs	**7**	**1217**	**1079**	**88.7**	**86.7**	**90.4**

## Data Availability

All data are included within the manuscript.

## Abbreviations

AEs: Adverse events
CI: Confidence interval
CR: Cure rate
DALYs: Disability-adjusted life years
ERR: Egg reduction rate
FECT: Formalin-ether concentration technique
*I* ^2^: Inverse Variance Index
MDA: Mass drug administration
NTD: Neglected tropical diseases
PRISMA: Preferred Reporting Items for Systematic Reviews and Meta-Analysis
RR: Risk ratio
SNNP: South Nations and Nationalities Peoples
WHO: World Health Organization.

## Supplementary Materials

Supplementary Materials Table S1: PRISMA checklist: the Preferred Reporting Items for Systematic Reviews and Meta-Analysis that indicates how the selection of relevant literatures and the systematic review were conducted.
